# Comparison of SARS-CoV-2 Test Positivity in NCAA Division I Student Athletes vs Nonathletes at 12 Institutions

**DOI:** 10.1001/jamanetworkopen.2021.47805

**Published:** 2022-02-09

**Authors:** Emily A. Schultz, Andrea Kussman, Alyssa Jerome, Geoffrey D. Abrams, Calvin E. Hwang

**Affiliations:** 1 Department of Orthopaedic Surgery, Stanford University School of Medicine, Stanford, California

## Abstract

**Importance:**

The COVID-19 pandemic initially led to the abrupt shutdown of collegiate athletics until guidelines were established for a safe return to play for student athletes. Currently, no literature exists that examines the difference in SARS-CoV-2 test positivity between student athletes and nonathletes at universities across the country.

**Objective:**

To identify the difference in risk of COVID-19 infection between student athlete and nonathlete student populations and evaluate the hypothesis that student athletes may display increased SARS-CoV-2 test positivity associated with increased travel, competition, and testing compared with nonathletes at their respective universities.

**Design, Setting, and Participants:**

In this cross-sectional analysis, a search of publicly available official university COVID-19 dashboards and press releases was performed for all 65 Power 5 National Collegiate Athletic Association (NCAA) Division I institutions during the 2020 to 2021 academic year. Data were analyzed at the conclusion of the academic year. Schools that released at least 4 months of testing data, including the fall 2020 football season, for student athletes and nonathlete students were included in the analysis. Power 5 NCAA Division I student athletes and their nonathlete student counterparts were included in the analysis.

**Exposure:**

Designation as a varsity student athlete.

**Main Outcomes and Measures:**

The main outcome was SARS-CoV-2 test positivity for student athletes and nonathlete students at the included institutions for the 2020 to 2021 academic year, measured as a relative risk for student athletes.

**Results:**

Among 12 schools with sufficient data available included in the final analysis, 555 372 student athlete tests and 3 482 845 nonathlete student tests were performed. There were 9 schools with decreased test positivity in student athletes compared with nonathlete students (University of Arkansas: 0.01% vs 3.52%; University of Minnesota: 0.63% vs 5.96%; Penn State University: 0.74% vs 6.58%; Clemson University: 0.40% vs 1.88%; University of Louisville: 0.75% vs 3.05%; Purdue University: 0.79% vs 2.97%; University of Michigan: 0.40% vs 1.12%; University of Illinois: 0.17% vs 0.40%; University of Virginia: 0.64% vs 1.04%) (*P* < *.*001 for each). The median (range) test positivity in these 9 schools was 0.46% (0.01%-0.79%) for student athletes and 1.04% (0.40%-6.58%) for nonathlete students. In 1 school, test positivity was increased in the student athlete group (Stanford University: 0.20% vs 0.05%; *P* < .001). Overall, there were 2425 positive tests (0.44%) among student athletes and 30 567 positive tests (0.88%) among nonathlete students, for a relative risk of 0.50 (95% CI, 0.48-0.52; *P* < .001). There was no statistically significant difference in student athlete test positivity between included schools; however, test positivity among nonathlete students varied considerably between institutions, ranging from 133 of 271 862 tests (0.05%) at Stanford University to 2129 of 32 336 tests (6.58%) at Penn State University.

**Conclusions and Relevance:**

This study found that in the setting of SARS-CoV-2 transmission mitigation protocols implemented by the NCAA, participation in intercollegiate athletics was not associated with increased SARS-CoV-2 test positivity. This finding suggests that collegiate athletics may be held without an associated increased risk of infection among student athletes.

## Key Points

**Question:**

Was participation in collegiate athletics associated with increased SARS-CoV-2 test positivity?

**Findings:**

In this cross-sectional study using data for 555 372 student athlete and 3 482 845 nonathlete student SARS-CoV-2 tests reported from 12 National Collegiate Athletic Association Division I institutions, participation in collegiate athletics was not associated with increased test positivity in student athletes compared with nonathlete students.

**Meaning:**

This finding suggests that collegiate athletics may be held safely in the COVID-19 pandemic without associated increases in test positivity among student athletes.

## Teaser

This cross-sectional study compares SARS-CoV-2 test positivity in college student athletes vs nonathlete students at 12 NCAA institutions.

## Introduction

Collegiate and professional athletics were shut down temporarily in spring 2020 owing to the COVID-19 pandemic. As various collegiate and professional organizations debated the resumption of athletic activities, 2 primary concerns came to the forefront: adverse cardiac sequelae postinfection and potentially increased viral transmission within the athletic footprint. While initial studies raised concerns of widespread cardiac involvement postinfection, larger follow-up studies found low rates of myocarditis and other cardiac abnormalities in young athletes.^1,2,3^ Strategies to mitigate disease transmission ranged from a complete bubble (in the National Basketball Association and Women’s National Basketball Association) to a hybrid bubble (in Major League Baseball) to local implementation of strict distancing, face covering, and testing protocols (in the National Football League and National Collegiate Athletic Association [NCAA]). However, the specific risk of transmission within a collegiate athletic team setting including meals, practice, travel, competition, and communal housing with these various protocols is unknown. Although there have been anecdotal reports of outbreaks of SARS-CoV-2 infection within athletic teams,^4^ these outbreaks have also been seen in other communal living settings. It is not known if collegiate student athlete infection rates are significantly higher than those of the general student or community population.^5,6,7^

In the NCAA’s Resocialization of Collegiate Sport document, specific guidelines on polymerase chain reaction (PCR) testing, training, physical distancing, and face coverings were implemented to mitigate the risk of transmission within athletics.^8^ Several studies have found these measures to be largely effective.^9,10,11^ Although these minimum NCAA guidelines were implemented across all institutions, there was still variation in the frequency of student athlete testing owing to additional individual university or county protocols on testing.^12^ Conversely, while most universities implemented a surveillance testing cadence for members of the university community (ie, students, faculty and staff, etc) using antigen or PCR tests, there was no minimum standard requirement to do so. To our knowledge, no study to date has looked at the association of participation in intercollegiate athletics with SARS-CoV-2 test positivity compared with those of the general university student population. This study examines test positivity of student athletes and nonathlete students at various universities during the 2020 to 2021 academic year to investigate if intercollegiate sport participation was associated with an increased risk of SARS-CoV-2 infection.

## Methods

This cross-sectional study used publicly available deidentified data and was approved by the Stanford University Institutional Review Board. Informed consent was exempted by the Stanford University Institutional Review Board based on the deidentified data. This study followed the Strengthening the Reporting of Observational Studies in Epidemiology (STROBE) reporting guideline.

Student athlete testing data was collected via an internet search across all Power 5 Division I institutions for publicly available SARS-CoV-2 testing data during the 2020 to 2021 academic year. When available, the total number of SARS-CoV-2 positive tests, test positivity, and total number of tests administered were recorded. For universities that presented sufficient student athlete testing data for analysis, the equivalent data for the undergraduate only population (if available) or overall student body with student athlete testing data removed were obtained for comparison. To be included, institutions had to have at least 4 months of data available for each group and include the fall football season.

### Statistical Analysis

Data for each group were obtained through official press releases or public data dashboards, and time frames were matched when possible. These raw numbers were used to calculate test positivity for each group within a university, dividing the number of positive SARS-CoV-2 tests by the total number of tests administered during the study period. The test proportions of positives were then used to calculate the relative risk of a student athlete positive test compared with a nonathlete at a given institution.

A 2-tailed *t* test was used to calculate differences between groups for each university and for the entire data set. A separate analysis was performed after combining test results of 4 universities that required regular surveillance testing (minimum once weekly) for the entire student body throughout the study period. All analyses were completed in RStudio statistical software version 1.1.456 (RStudio) using a 2-sided level of significance of *P* = .05. Data were analyzed at the conclusion of the academic year.

## Results

**Figure 1.**
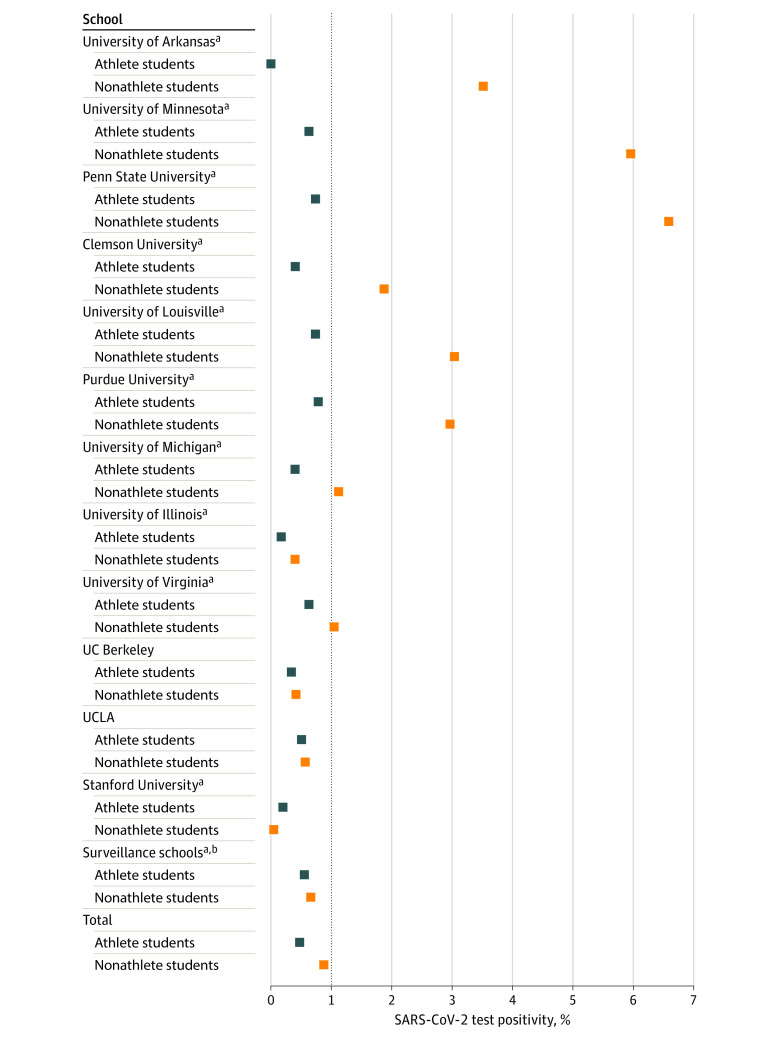
SARS-CoV-2 Test Positivity in Student Athletes and Nonathlete Students UC Berkeley indicates University of California, Berkeley; UCLA, University of California, Los Angeles. ^a^*P* < .001. ^b^Schools that performed once or twice weekly surveillance testing for nonathlete students throughout the 2020 to 2021 academic year.

**Figure 2.**
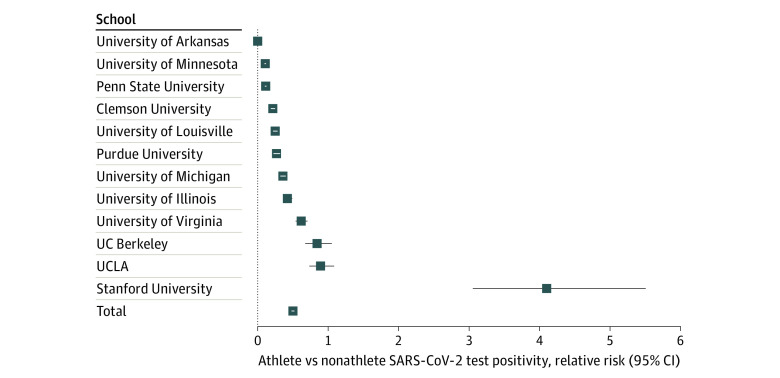
Student Athlete Test Positivity Relative Risk UC Berkeley indicates University of California, Berkeley; UCLA, University of California, Los Angeles. Boxes indicate relative risk; whiskers, 95% CI.

Among more than 4 million tests included in the study, 555 372 tests were among student athletes, with 2425 positive results (0.44%), and 3 482 845 tests were among nonathlete students, with 30 567 positive results (0.88%). Of 65 Power 5 schools, 13 schools had publicly available information on athlete and nonathlete test positives, total number of tests, and positivity (Table). One school was excluded given that the time period of data available did not meet the minimum duration determined in inclusion criteria. The remaining 12 schools were included in the analysis, 9 of which had statistically significantly decreased test positivity in the student athlete population compared with the nonathlete student population (University of Arkansas: 0.01% vs 3.52%; University of Minnesota: 0.63% vs 5.96%; Penn State University: 0.74% vs 6.58%; Clemson University: 0.40% vs 1.88%; University of Louisville: 0.75% vs 3.05%; Purdue University: 0.79% vs 2.97%; University of Michigan: 0.40% vs 1.12%; University of Illinois: 0.17% vs 0.40%; University of Virginia: 0.64% vs 1.04%) (*P* < *.*001 for each) (Box). The median (range) test positivity in these 9 schools was 0.46% (0.01%-0.79%) for student athletes and 1.04% (0.40%-6.58%) for nonathlete students (Figure 1). The relative risk for student athletes at these schools vs nonstudent athletes ranged from 0.002 (95% CI, 0.0005-0.01) for the University of Arkansas to 0.61 (95% CI, 0.54-0.70) for the University of Virginia (Figure 2). Of the remaining 3 schools, there was no statistically significant difference in test positivity at 2 of them, and 1 institution had a statistically significantly increased test positivity among student athletes (Stanford University: 0.20% vs 0.05%; relative risk, 4.08 [95% CI, 3.04-5.48]; *P* < .001). Overall, the relative risk for student athletes vs nonathlete students was 0.50 (95% CI, 0.48-0.52; *P* < .001) (Figure 2).

**Table.** Test Positivity by School

University	Student athletes			Nonathlete students			*P* value
	Total tests, No.	Total positive tests, No.	Positivity, %	Total tests, No.	Total positive tests, No.	Positivity, %	
University of Arkansas^a^	28 500	2	0.01	40 210	1415	3.52	<.001
University of Minnesota^a^	64 832	409	0.63	23 763	1417	5.96	<.001
Penn State University^a^	56 812	420	0.74	32 336	2129	6.58	<.001
Clemson University^b^	47 275	190	0.40	349 978	6589	1.88	<.001
University of Louisville^a^	34 957	261	0.75	67 530	2059	3.05	<.001
Purdue University^a^	18 688	147	0.79	19 358	574	2.97	<.001
University of Michigan^b^	86 000	344	0.40	440 386	4946	1.12	<.001
University of Illinois^c^	105 000	175	0.17	1 385 109	5490	0.40	<.001
University of Virginia^a^	34 583	221	0.64	271 434	2826	1.04	<.001
UC Berkeley^c^	25 000	86	0.34	196 979	809	0.41	.13
UCLA^c^	20 648	104	0.50	383 900	2180	0.57	.25
Stanford University^c^	33 077	66	0.20	271 862	133	0.05	<.001
Schools with regular testing^c^	183 725	431	0.23	2 237 850	8612	0.38	<.001
Overall total	555 372	2425	0.44	3 482 845	30 567	0.88	<.001

Abbreviations: UC Berkeley, University of California, Berkeley; UCLA, University of California, Los Angeles.

^a^ University did not require regular surveillance testing for nonathlete students.

^b^ University had weekly surveillance testing in spring 2021 semester only for nonathlete students.

^c^ University had weekly or twice weekly surveillance testing for nonathlete students throughout the 2020 to 2021 academic year.

Box. **Surveillance Testing Schedule for Nonathlete Students**

**No Regular Surveillance Testing**

Penn State University
Purdue University
University of Arkansas
University of Louisville
University of Minnesota
University of Virginia

**Once Weekly Starting Spring 2021**

Clemson University
University of Michigan

**Once or Twice Weekly Throughout 2020-2021 Year**

Stanford University
University of California, Berkeley
University of California, Los Angeles
University of Illinois

While 4 schools required weekly or twice weekly surveillance testing for nonathlete students throughout the 2020 to 2021 academic year, 2 schools implemented weekly surveillance testing only in the spring 2021 semester. The remaining 6 schools did not have any regular surveillance testing for nonathlete students, although some had on-arrival or random surveillance testing. (Box) All schools offered testing for nonathlete students with symptoms. All student athlete surveillance testing had to meet minimum standards determined by the NCAA, including at least weekly PCR testing in athletes participating in “high risk of transmission” sports (eg, football, basketball, hockey, and wrestling).^8^

To mitigate the differences in testing frequency between student athletes and nonathlete students, a separate analysis was conducted looking only at schools that had regular weekly or twice weekly surveillance testing for all nonathlete students. In these 4 schools, the student athlete relative risk was 0.61 (95% CI, 0.55-0.67; *P* < .001). There was a lack of heterogeneity in student athlete test positivity, with no statistically significant differences among included schools. However, there was wide variation in nonathlete test positivity, ranging from 133 of 271 862 tests (0.05%) at Stanford University to 2129 of 32 336 tests (6.58%) at Penn State University. The 4 schools that required weekly or twice weekly surveillance testing had the 4 lowest test positivities in the cohort.

## Discussion

In this cross-sectional study, SARS-CoV-2 test positivity among student athletes did not vary by institution. This could be associated with implementation of surveillance and containment strategies across the NCAA. The 3 schools that did not have statistically significantly decreased student athlete test positivity compared with nonathlete student positivity were all located in California. Stringent public health guidelines at the local level could have been associated with this finding. Furthermore, all 3 had required weekly or twice weekly testing for the entire academic year. They had similar student athlete test positivity as the rest of the schools analyzed, but some of the lowest nonathlete student test positivity, possibly associated with these public health restrictions, as well as the frequency of regular surveillance testing for nonathlete students.

The specific mitigation protocols implemented and the frequency of surveillance testing varied widely between student athlete and nonstudent athlete populations and among universities. By NCAA standards, all in-season student athletes participating in high contact risk sports were tested a minimum of once per week, but out-of-season student athletes or those in low or medium contact risk sports were required to test once per month. Several institutions exceeded these standards and tested all student athletes and nonathlete students at least weekly, while others did not have any required surveillance testing for nonathlete students. Increased rates of surveillance testing in individuals without symptoms could be associated with decreased test positivity if these individuals would not otherwise have been testing; thus, among institutions that tested student athletes more frequently than nonathlete students, one could expect a decreased proportion testing positive. There also appeared to be an inverse association in testing frequency with positivity in nonathlete students. Institutions that implemented regular surveillance testing for nonathlete students had decreased test positivity compared with institutions that did only on-arrival, random, or symptomatic testing, which is consistent with repetitive testing in many other settings. In fact, the 4 universities that required weekly or twice weekly surveillance testing for nonathlete students throughout the academic year had the lowest nonathlete student test positivity of the cohort. Conversely, there are also several factors which could be associated with increased test positivity among student athletes. First, student athletes were traveling for competition regularly and may have been in close contact with a larger number of individuals outside their athletic teams and universities compared with nonathlete students. Moreover, student athletes were also frequently in close contact with their teammates during practice and competitions, particularly in sports designated as high risk for transmission; in these settings, social distancing and face coverings were not mandated by the NCAA or universities. These factors could be associated with increased risk of SARS-CoV-2 infection in student athletes.

### Limitations

Despite being one of the first studies, to our knowledge, comparing test positivity in collegiate student athletes with nonathlete students, there are several limitations to this study. Most significantly, only 12 of 65 Power 5 institutions had publicly available testing data for analysis. It is unknown if these 12 schools are representative of the overall collegiate student athlete and nonathlete student population, particularly given that there has been significant geographic variability in SARS-CoV-2 infection rates and public health measures across the country. There may be a selection bias present in those universities that opted to make their data publicly available. As we noted previously, surveillance test frequency varied significantly among institutions and student populations, which was likely associated with test positivity. It is also possible that infection rates may have varied among sports; however, sport-specific data were not available for analysis. Given that the definition of student athlete was left up to the reporting institution, it is unknown if all student athletes were actively participating in sport during the period analyzed. Another important consideration is the association of vaccinations with positivity rates. Because the study analyzed tests from the 2020 to 2021 academic year, vaccinations became more widely available during the latter part of the study period, and the association of vaccination with positivity rates in team and university settings in the future is unclear. Moreover, the study period took place prior to the rise of the SARS-CoV-2 Delta and Omicron variants, and the association of this and future variants with test positivity in collegiate athletic settings is still to be determined.

## Conclusions

This study found that varsity collegiate student athletes did not have increased risk of SARS-CoV-2 infection compared with nonathlete students, and at many institutions, they had a decreased risk. The COVID-19 mitigation strategies implemented by the NCAA and individual universities may have been associated with these results. However, the association of future SARS-CoV-2 variants and more widespread vaccination with positivity outcomes is unknown.
